# Methylmercury Concentration in Fish and Risk-Benefit Assessment of Fish Intake among Pregnant versus Infertile Women in Taiwan

**DOI:** 10.1371/journal.pone.0155704

**Published:** 2016-05-17

**Authors:** Hsing-Cheng Hsi, You-Wen Hsu, Tien-Chin Chang, Ling-Chu Chien

**Affiliations:** 1 Graduate Institute of Environmental Engineering, National Taiwan University, Taipei, Taiwan; 2 Institute of Environmental Engineering and Management, National Taipei University of Technology, Taipei, Taiwan; 3 School of Public Health, Taipei Medical University, Taipei, Taiwan; Chinese Academy of Sciences, CHINA

## Abstract

This study examined methylmercury (MeHg) concentrations in fish, the daily MeHg exposure dose, and the risk–benefit of MeHg, ω-3 polyunsaturated fatty acid (ω-3 PUFA), docosahexaenoic acid (DHA), and eicosapentaenoic acid (EPA) related to fish intake among pregnant and infertile women in Taiwan. The measured MeHg concentrations in fish did not exceed the Codex guideline level of 1 mg/kg. Swordfish (0.28 ± 0.23 mg/kg) and tuna (0.14 ± 0.13 mg/kg) had the highest MeHg concentrations. The MeHg concentration in the hair of infertile women (1.82 ± 0.14 mg/kg) was significantly greater than that of pregnant women (1.24 ± 0.18 mg/kg). In addition, 80% of infertile women and 68% of pregnant women had MeHg concentrations in hair that exceeded the USEPA reference dose (1 mg/kg). The MeHg concentrations in hair were significantly and positively correlated with the estimated daily MeHg exposure dose. Based on the risk–benefit evaluation results, this paper recommends consumption of fish species with a low MeHg concentration and high concentrations of DHA + EPA and ω-3 PUFA (e.g., salmon, mackerel, and greater amberjack).

## Introduction

Mercury (Hg) is a persistent element that bioaccumulates in humans and wildlife. It may influence the endocrine system, which could lead to a decrease in ovarian function, an irregular menstrual cycle, abortion, and infertility [1–6]. Methylmercury (MeHg) has a lipophilic characteristic and readily bioaccumulates in exposed organisms, after which it penetrates the blood–brain barrier, causing severe harm to the central nervous system [7, 8]. Recent evidence indicates that maternal low-level MeHg exposure may have adverse effects on fetal birth outcomes and growth [9]. Humans are exposed to MeHg through fish intake because Hg can transform into MeHg in aquatic environments. According to recent studies, fish intake is considered a major pathway of exposure to MeHg. McDowell et al. [10] found that women and children in the United States who reported consuming fish frequently had 3- (0.38 vs. 0.11 mg/kg) and 2-fold (0.16 vs. 0.08 mg/kg) hair Hg levels, respectively, compared with those who did not consume fish. A study from Italy indicated that hair Hg levels increased by 2-fold for people consuming more fish (5–6 meals per week) compared with those who consumed less fish (i.e., less than 5 meals per week) [11].

In Hong Kong, a study found that greater blood Hg concentration in infertile women may interfere with their endocrine system and induce deleterious effects on reproduction [12]. Cole et al. [13] determined that couples with a maternal blood Hg concentration > 1.2 μg/L (0.24 ppm in hair) may require more time to become pregnant. In another two of our earlier studies [14, 15], the average maternal blood Hg concentration was 9.1 ± 0.40 μg/L, and 89% of mothers had a concentration exceeding the US National Research Council (USNRC) recommended value of 5.8 μg/L. There were 53% of women of childbearing age with Hg concentrations in hair exceeding the US Environmental Protection Agency (USEPA) reference dose of 1 mg/kg. Both of these two reference values were adopted by USNRC and USEPA with a purpose to avoid from Hg influence on the fetus neurodevelopment. Additionally, the fish consumption rate was found to be significantly correlated with the maternal blood and Hg concentrations in hair of pregnant women and women of childbearing age in Taiwan. Notably, data from the Department of Statistics, Ministry of the Interior, Taiwan showed that the infertility prevalence is approximately 15% in Taiwan couples [16]. Moreover, postnatal MeHg exposure could reduce expressive language performance in children who cannot make independent dietary decisions and defer more to their mothers [17]. The Nutrition and Health Surveys in Taiwan investigated changes in dietary habits for the 1993–1996 and 2005–2008 periods and observed an increase in fish intake [18]. Fish intake is thus a critical dietary source among the Taiwan population, which may be exposed to both MeHg and the intake of ω-3 polyunsaturated fatty acid (ω-3 PUFA), docosahexaenoic acid (DHA), and eicosapentaenoic acid (EPA). In addition, ω-3 PUFA and DHA have been known to increase the gestation duration and birth weight, and to reduce the risk of early preterm delivery [19].

The aforementioned results suggest that enhanced comprehension of MeHg concentrations in fish, the daily MeHg exposure dose, and the risks and benefits of MeHg, ω-3 PUFA, and DHA + EPA intake as related to fish intake is critical for women of childbearing age. In the present paper, we systematically analyze MeHg concentrations in fish and hair to examine the daily MeHg exposure dose among pregnant and infertile women in Taiwan. We further investigate the benefits of ω-3 PUFA and DHA + EPA as well as the risks of MeHg related to fish intake. According to the results of the risk–benefit assessment of fish intake, we can provide a useful recommendation on the species and quantity of fish consumption for women and reduce MeHg exposure for fetuses.

## Materials and Methods

### Participants and sample collection

For this study, 224 women residing in Northern and Central Taiwan from August 2007 to May 2010 were recruited. We categorized women as infertile or pregnant according to their reproductive status, which was confirmed by 3 gynecologists. Infertile women were defined as those who presented with a complaint regarding difficulty conceiving after 1 year of normal sexual activity with the intention of becoming pregnant [20]. The demographic characteristics and dietary information of the participants were obtained using a structured questionnaire. All of the participants provided written informed consent prior to enrollment. The Institutional Review Board at Taipei Medical University (approval number: P950045) and the Investigational Review Board of Taiwan Adventist Hospital (TAIRB number: 989801A) have approved this study.

Hair samples of approximately 2 cm in length were collected from the occipital area of the scalp by using clean stainless steel surgical scissors. The hair samples were placed, sealed, and stored in clean polythene bags until analysis. To assess the daily MeHg exposure dose through fish intake, the 10 most popular species of fish (30 samples, with 3 samples for each species) were determined and selected from the questionnaire. The fish samples were divided into the following 3 categories according to the Fisheries Agency database: (1) carnivorous: mackerel (*Scomber australasicus*), tilapia (*Oreochromis mossambicus*), hairtail (*Trichiurus lepturus*), salmon (*Oncorhynchus mykiss*), greater amberjack (*Seriola dumerili*), cod (*Gadus macrocephalus*), tuna (*Thunnus alalunga*), and swordfish (*Xiphias gladius*); (2) omnivorous: milkfish (*Chanos chanos*); and (3) filter feeding: anchovy (*Stolephorus commersonnii*) [21]. Fish samples were randomly obtained from March to June in 2014 at fish markets, supermarkets, and traditional markets in Taipei City. The samples were placed in clean polyethene bags and transferred to the laboratory immediately after collection. The fish muscle was cleaned, ground with pestle, homogenized, and stored at −18°C for subsequent analysis.

### Hair and fish sample analysis

The MeHg concentrations in hair and fish were analyzed through the Brooks Rand BRL Method BR-0011 and the USEPA Method 1630, with minor modifications. Hair samples were treated with a neutral detergent by performing sonication for 30 min, rinsed 3 times with deionized (d.i.) water, and then dried in an oven at 37°C for 24 h. Details of the analytical method are available in our previous study [17]. The 1.0-g fish samples were transferred to Teflon vials, to which 2 mL of 25% KOH/methanol was added and heated at 75°C for 5.5 h. After being cooled to room temperature, 10 mL of CH_2_Cl_2_ and 2 mL of HCl were further added to the samples and violently shaken for 30 min. After being filtrated using a 1PS filter, the solution was purged for 1 h after the addition of d.i. water. The aliquot was diluted to 100 mL with d.i. water in a Teflon vial. The MeHg concentration was analyzed using a MERX integrated automated MeHg analyzer (Brooks Rand, USA). A 100-mL aliquot from the hair samples and a 1-mL aliquot from the fish samples were used for analysis. A sodium acetate buffer (300 mL) and 1.0% NaBEt_4_ (40 μL) were added into the aliquot prior to analysis.

Aliquots of 10 mL and 100 μL from the standard reference materials were analyzed to confirm the quality of the hair and fish test sets, respectively. IAEA-085 MeHg, total Hg, and other trace elements in human hair from the International Atomic Energy Agency as well as certified reference material BCR-465 tuna fish were used to ensure the precision and accuracy of the hair and fish analyses. The average recovery rate was 94.7%, and each sample was analyzed in triplicate. The precision (coefficient of variation) and accuracy were 4.27% and 89.2%, respectively.

### Daily MeHg exposure dose

Information on fish intake was obtained using a structured questionnaire. The individual daily MeHg exposure dose was assessed using Eq 1 from the US Environmental Protection Agency [22], which was modified for this study.

$${\text{E}}_{\text{m}}=\frac{{\text{C}}_{\text{m}}\times \text{IR}}{\text{BW}}=\frac{\sum_{\text{j}=1}^{\text{n}}\left({\text{C}}_{\text{mj}}\times {\text{IR}}_{\text{j}}\right)}{\text{BW}}=\frac{\left(\frac{\sum_{\text{j}=1}^{\text{n}}\left({\text{C}}_{\text{mj}}\times {\text{IR}}_{\text{j}}\right)}{\sum_{\text{j}=1}^{\text{n}}{\text{IR}}_{\text{j}}}\right)\left(\sum_{\text{j}=1}^{\text{n}}\text{I}{\text{R}}_{\text{j}}\right)}{{\text{BW}}_{\text{i}}}$$ (1)

where E_m_ = the dietary MeHg exposure dose from fish (μg/kg/d); Cm = the MeHg concentration in fish (mg/kg wet wt.); IR = the ingestion rate of fish (g/d); C_mj_ = the MeHg concentration in species j fish (mg/kg wet wt.); IR_j_ = the ingestion rate of species j fish (g/d); and BW_i_ = the individual body weight (kg).

### Risk–benefit assessment of specific fish intake

Eq 2 was solved to estimate the hazard quotient (HQ) for women of childbearing age [23]. The HQ was the ratio between the daily MeHg exposure dose and the reference dose.

$$\text{HQ}=\frac{{\text{C}}_{\text{mj}}\times {\text{IR}}_{\text{ave}}}{\text{RfD}\times \text{BW}}$$ (2)

where C_*mj*_ = the MeHg concentration in species j fish (mg/kg wet wt.); IR_ave_ = the average ingestion rate of ingesting fish (30 g/d); BW = body weight (56 kg was used in this study); and RfD = the reference dose of the US Environmental Protection Agency (i.e., 0.1 μg/kg/d).

The proportion of the desirable dose consumed for the DHA + EPA and ω-3 PUFA concentrations of specific fish was assessed using Eq 3:

$${\text{D}}_{\text{desire}}=\frac{{\text{C}}_{\text{FAj}}\times IR_{ave}}{RDI_{FAj}}$$ (3)

where D_desire_ = the proportion of the desirable dose; C_FAj_ = DHA + EPA or ω-3 PUFA concentrations in species j fish (mg/g wet wt.); IR_ave_ = the average ingestion rate of fish (30 g/d); and RDI_FA_ = the recommended daily intake of DHA + EPA or ω-3 PUFA concentrations (mg/d). For nonpregnant and nonlactating women, a minimum intake of 250 mg/d of DHA + EPA was recommended by the Food and Agriculture Organization of the United Nations as well as the World Health Organization [24]. For adults, an approximate minimum intake of 1000 mg/d of ω-3 PUFA was recommended by the Ministry of Health, Labour, and Welfare, Japan, for optimal health [25].

Eq 4 was solved to predict the allowable daily intake of fish for women of childbearing age according to the MeHg concentration of fish species (wet wt.):

$${\text{ADI}}_{\text{j}}=\frac{\text{BW}\;\times \;\text{RfD}}{{\text{C}}_{\text{mj}}}$$ (4)

where ADI_j_ = the allowable daily intake of species j fish (g/d); BW = body weight (56 kg was used for this study); RfD = the reference dose of the US Environmental Protection Agency (i.e., 0.1 μg/kg/d); and C_mj_ = MeHg concentration in species j fish (mg/kg wet wt.).

### Statistical analysis

The distribution of continuous variables (i.e., age, height, and weight) are expressed as the mean ± standard deviation. The nonparametric Wilcoxon rank-sum test was conducted to compare the parameters that were not normally distributed. The chi-square test and Fisher exact test were performed to compare the categorical variables between pregnant and infertile women. The Spearman rank correlation coefficient was used to compare the relationship between MeHg concentrations in hair and the daily MeHg exposure dose. All statistical analyses were conducted using SPSS (version 17.0) for Windows. The level of significance in a 2-sided test was considered to be *P* < 0.05.

## Results and Discussion

In this study, the infertile women (34.4 ± 3.7) were on average older than the pregnant women (31.6 ± 4.9) (*P* < 0.0001) (Table 1). The frequency of Chinese herbal medicine use and sashimi intake in the infertile group was greater than that in pregnant women (*P* < 0.05). The geometric mean (GM) MeHg concentration in the hair of the pregnant and infertile women was 1.24 ± 0.18 mg/kg and 1.82 ± 0.14 mg/kg, respectively. The MeHg concentrations in the hair of the infertile women were significantly higher than those in the hair of the pregnant women (*P* = 0.01). In addition, 80% of the infertile women and 68% of the pregnant women had MeHg concentrations in hair that exceeded the US Environmental Protection Agency reference dose of 1 mg/kg. We collected 2-cm hair samples from the occipital area of the scalp, which indicated the previous 1–2 months of exposure as suggested by Díez et al. [11]; 32 maternal hair samples after delivery (including the first, second, and third trimester) were collected. Significant differences in Hg concentrations in hair among the 3 trimesters (*P* = 0.54, S1 Table) were not found. Thus, hair could be used as a suitable indicator of long-term Hg exposure because Hg exposure is considered being constant.

**Table 1:** Demographic characteristics and MeHg concentrations in the hair of study participants.

	Pregnant women	Infertile women	*P* value
	(*n* = 62)	(*n* = 162)	
Age (y) ^a^	31.6 ± 4.9	34.4 ± 3.7	<0.0001 ^b^
Height (cm) ^a^	160.4 ± 5.4	160.0 ± 5.3	0.840 ^b^
Weight (kg) ^a^	55.9 ± 8.2	55.4 ± 11.4	0.567 ^b^
Education level			0.003 ^c^
Senior high school	16 (25.8)	14 (8.7)	
College	38 (61.3)	114 (70.8)	
Graduate	8 (12.9)	33 (20.5)	
Occupational exposure			0.558 ^d^
Yes	5 (8.3)	10 (6.2)	
No	55 (91.7)	151 (93.8)	
Amalgam fillings			0.025 ^c^
No	24 (40.0)	35 (22.7)	
1–3	26 (43.4)	67 (43.5)	
4–6	8 (13.3)	36 (23.4)	
**≥**7	2 (3.3)	16 (10.4)	
Alcohol consumption			0.002 ^c^
Yes	3 (5.0)	40 (24.8)	
No	57 (95.0)	121 (75.2)	
Chinese herbal medicine use			<0.001 ^c^
Never	15 (24.2)	7 (4.4)	
<1 time per month	31 (50.0)	71 (44.7)	
≥1 time per month	16 (25.8)	81 (50.9)	
Smoking			0.296 ^d^
Yes	1 (1.7)	10 (6.2)	
No	59 (98.3)	151 (93.8)	
Fresh fish intake			0.387 ^c^
≤1 meal per week	23 (37.1)	74 (46.0)	
1–2 meals per week	30 (48.4)	62 (38.5)	
**≥**3 meals per week	9 (14.5)	25 (15.5)	
Sashimi intake			<0.05 ^d^
Never	30 (48.4)	49 (30.8)	
1–3 meals per month	31 (50.0)	106 (66.7)	
≥1–2 meals per week	1 (1.6)	4 (2.5)	
MeHg concentration (mg/kg wet wt.)^e^	1.24 ± 0.18	1.82 ± 0.14	0.01 ^b^

^a^ Mean ± standard deviation

^b^ Wilcoxon rank-sum test

^c^ Chi-square test

^d^ Fisher exact test

^e^ Geometric mean ± standard error

**Fig 1:**
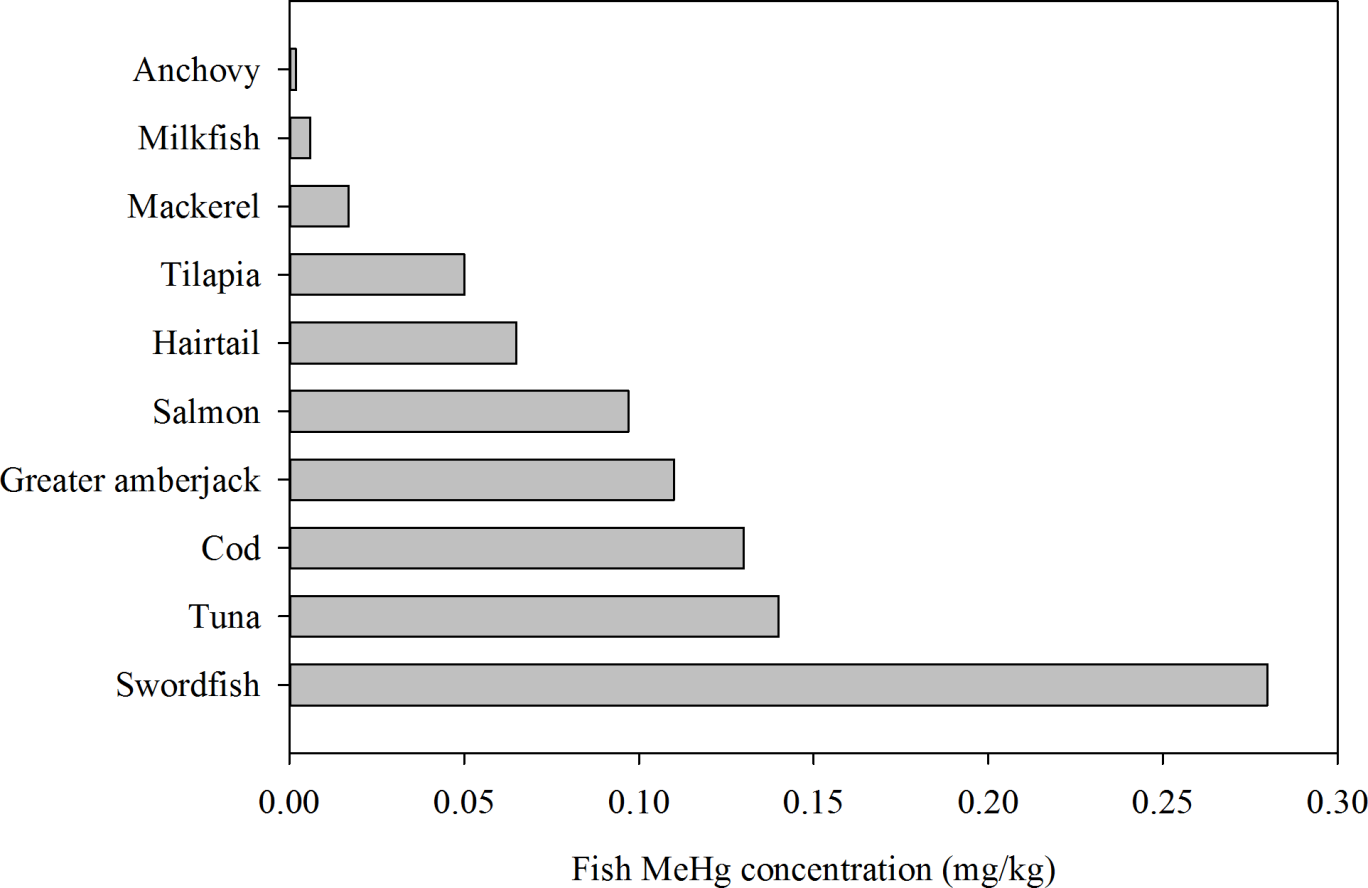
Average MeHg concentrations in the 10 most popular fish muscles for women of childbearing age. The fish was acquired from markets in Taiwan (*n* = 30 samples, 3 samples for each species).

The MeHg concentration distributions of the fish that were frequently consumed by the women are summarized in Fig 1 and S2 Table. The highest MeHg concentrations were in swordfish (*X*. *gladius*: 0.28 ± 0.23 mg/kg wet wt.) and tuna (*T*. *alalunga*: 0.14 ± 0.13 mg/kg wet wt.). Karjalainen et al. [26] reported that the MeHg concentrations in tuna, cod, and salmon in Finland ranged from 0.19–0.31, 0.038–0.056, and 0.01–0.10 mg/kg wet wt., respectively. A recent study in North America reported MeHg concentrations in tuna (0.49 ± 0.07 mg/kg wet wt.), cod (0.14 ± 0.09 mg/kg wet wt.), salmon (0.014 ± 0.013 mg/kg wet wt), and tilapia (0.016 ± 0.012 mg/kg wet wt.) [27]. Levels of MeHg in Tuna were 0.78 ± 0.91 mg/kg wet wt. and 0.14 ± 0.02 mg/kg wet wt. in Portuguese and Spain, respectively [28, 29]. In Hong Kong, the MeHg concentration in tilapia ranged from 0.009 to 0.042 mg/kg wet wt. [30]. Therefore, the fish MeHg concentrations in the muscle of the same species of fish from the selected Taiwan markets were found to be higher than those collected in Finland and Hong Kong, but lower than those in North America, Portuguese, and Spain. Our results also revealed that the MeHg concentrations in fish varied according to diet in the following order: carnivorous fish (0.11 ± 0.13 mg/kg wet wt.) > omnivorous fish (0.006 ± 0.01 mg/kg wet wt.) > filter-feeding fish (0.002 ± 0.001 mg/kg wet wt.). Several earlier studies further indicated that the feeding habits and trophic positions of fish could affect the variability in MeHg concentrations in fish muscle [31–36]. Carnivorous fish were found to have the highest MeHg concentration in muscle (average range: 0.022–1.179 mg/kg dry wt.) [36], which was consistent with the findings of the present study. Notably, the MeHg concentrations of fish in this study did not exceed the Codex guideline level of 1 mg/kg [37].

Several factors can affect the concentration of MeHg in hair. We explored the correlation between age, fish and sashimi consumption, and MeHg concentration in the hair of our participants (Table 2). Women aged ≥ 35 years exhibited greater hair MeHg concentrations compared with those aged < 35 years (*P* = 0.003). According to their fish consumption rate, we divided the participants into 3 groups (i.e., < 1 meal per week, 1–2 meals per week, and 3 meals per week) to distinguish the differences in hair MeHg concentrations. Hair MeHg concentrations in the groups who consumed fresh fish for 1–2 meals per week and > 3 meals per week were marginally significantly higher than those for groups consuming fish for < 1 meal per week (*P* = 0.053). The MeHg concentrations in hair obtained for this study were comparable to those of mothers in Paris, France (mean = 1.37 mg/kg), South Korea (mean = 0.91 mg/kg), and Porto Velho, Brazil (median = 1.3 mg/kg) [38–40]. Shao et al. [41] found that the MeHg concentration in hair increased with age. In our previous study, the average Hg concentration in hair was 1.73 ± 2.12 mg/kg for women of childbearing age in Taiwan, including college students, the public, and dental as well as medical workers [14]. We also found that the MeHg concentrations in the hair of infertile women in this study were substantially greater than those of female college students (GM = 0.73 mg/kg) and the general public (males and females; GM = 0.82 mg/kg) in Taiwan. In addition, the MeHg concentrations in hair were positively correlated with the fish consumption frequency. A similar pattern was observed previously because those who consumed more sashimi (≥ 1–2 meals per week) had the highest MeHg concentrations in hair (3.68 ± 0.62 mg/kg). Díez et al. [11] indicated that those who consumed less fish (less than 5 meals per week) had a 0.5-fold hair MeHg level compared with those who consumed more fish (5–6 meals per week). Salehi and Esmaili-Sari [42] reported that elevated hair Hg concentration was positively correlated with frequent fish consumption in pregnant women.

**Table 2:** Summary of MeHg concentrations in hair, categorized by age and types of fish intake, including fresh fish and sashimi intake.

		Hair MeHg concentration (mg/kg)				
	n	Geomean ± SE	Median	Max	Min	*P* value
Age (<35 y)	138	1.42 ± 0.14	1.64	10.5	0.01	0.003 ^a^
Age (≥ 35 y)	86	2.07 ± 0.19	2.16	7.44	0.016	
Fresh fish intake						0.053 ^b^
<1 meal per week	97	1.32 ± 0.14	1.69	6.37	0.01	
1–2 meals per week	92	1.87 ± 0.19	1.82	10.5	0.03	
≥3 meals per week	34	2.07 ± 0.32	2.05	7.63	0.27	
Sashimi intake						0.002 ^b^
Never	79	1.29 ± 0.16	1.56	7.44	0.01	
1–3 meals per month	137	1.88 ± 0.15	1.96	10.5	0.01	
≥1–2 meals per week	5	3.68 ± 0.62	4.34	5.58	2.16	

^a^ Wilcoxon rank-sum test

^b^ Kruskal–Wallis test

**Fig 2:**
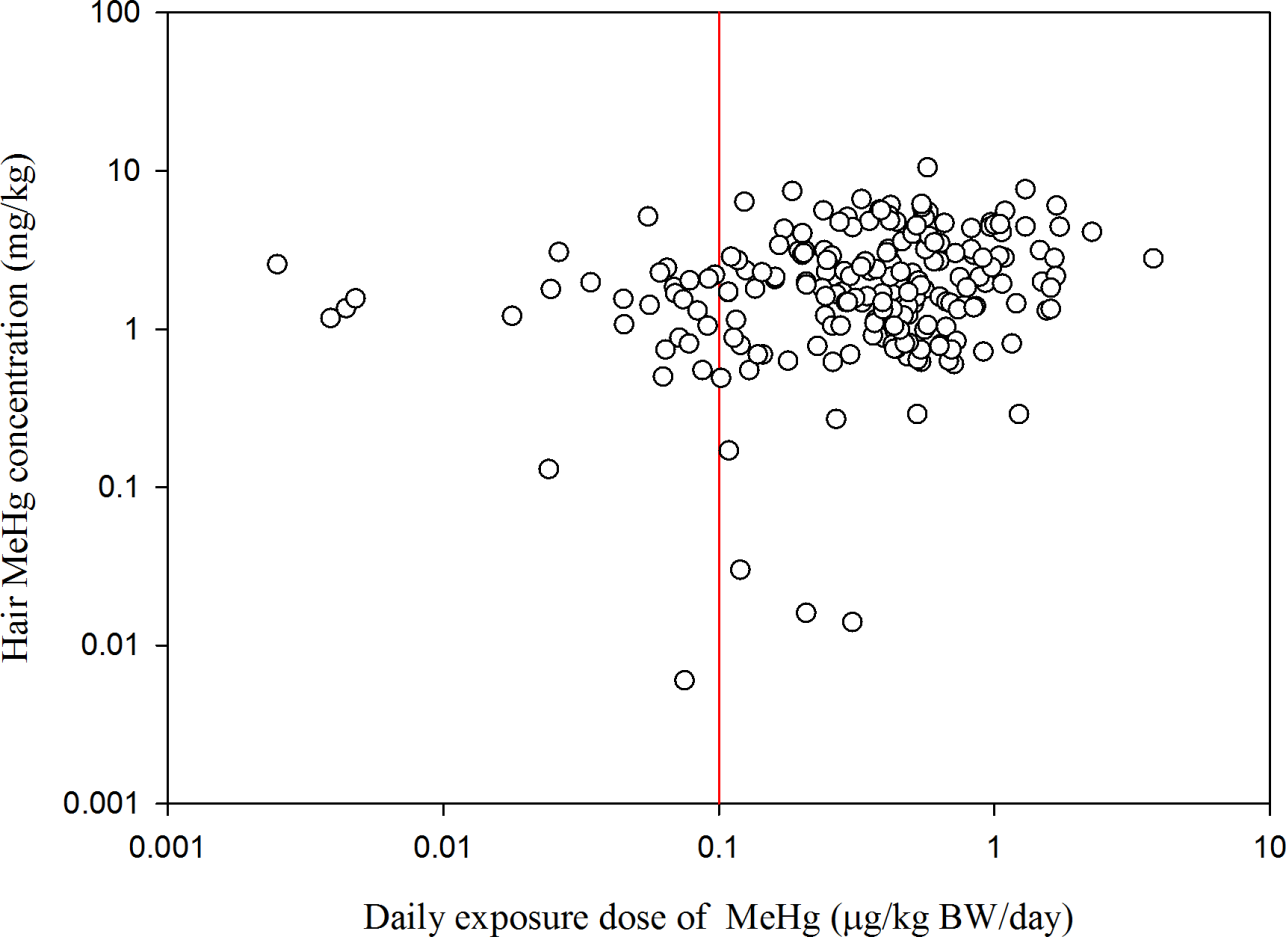
Relationship between MeHg concentrations in hair and daily MeHg exposure dose through fish intake.

The relationship between the MeHg concentration in hair and the daily MeHg exposure dose through fish intake is illustrated in Fig 2. We observed a significantly positive correlation between hair MeHg concentration and the daily MeHg exposure dose (r = 0.24, *P* < 0.01). The calculated daily MeHg exposure dose was 0.34 ± 0.34 μg/kg/d for pregnant women and 0.51 ± 0.49 μg/kg/d for infertile women. Our findings also showed that the daily MeHg exposure dose for infertile women was significantly greater than that for pregnant women (*P* < 0.01). Based on the reference dose of the US Environmental Protection Agency for MeHg of 0.1 μg/kg/d, 90.9% and 76.7% of the daily MeHg exposure dose estimates exceeded the reference dose for the infertile and pregnant women, respectively. When the daily MeHg exposure dose exceeds the reference dose over a lifetime, it may pose a risk for sensitive subgroups (i.e., fetuses and children). Shao et al. [41] reported that the estimated MeHg exposure dose for adults (aged between 24 and 54 years) through fish intake was 0.03 to 0.4 μg/kg/d in Pearl River Delta, mainland China. They also observed that the MeHg concentration in hair was significantly correlated with the daily MeHg exposure dose through fish consumption (r = 0.48, *P* < 0.01). This may be explained by the possibility that the participants who consumed more fish could have had a higher MeHg concentration in hair as well as a greater daily MeHg exposure dose. In Hong Kong, the estimated daily MeHg intake for 36% of adults and 51% of children who consumed fish obtained from markets exceeded the reference dose of the US Environmental Protection Agency; nevertheless, regarding the bioaccessibility of MeHg, only 9% of children had a daily MeHg intake that exceeded the reference dose [30].

**Fig 3:**
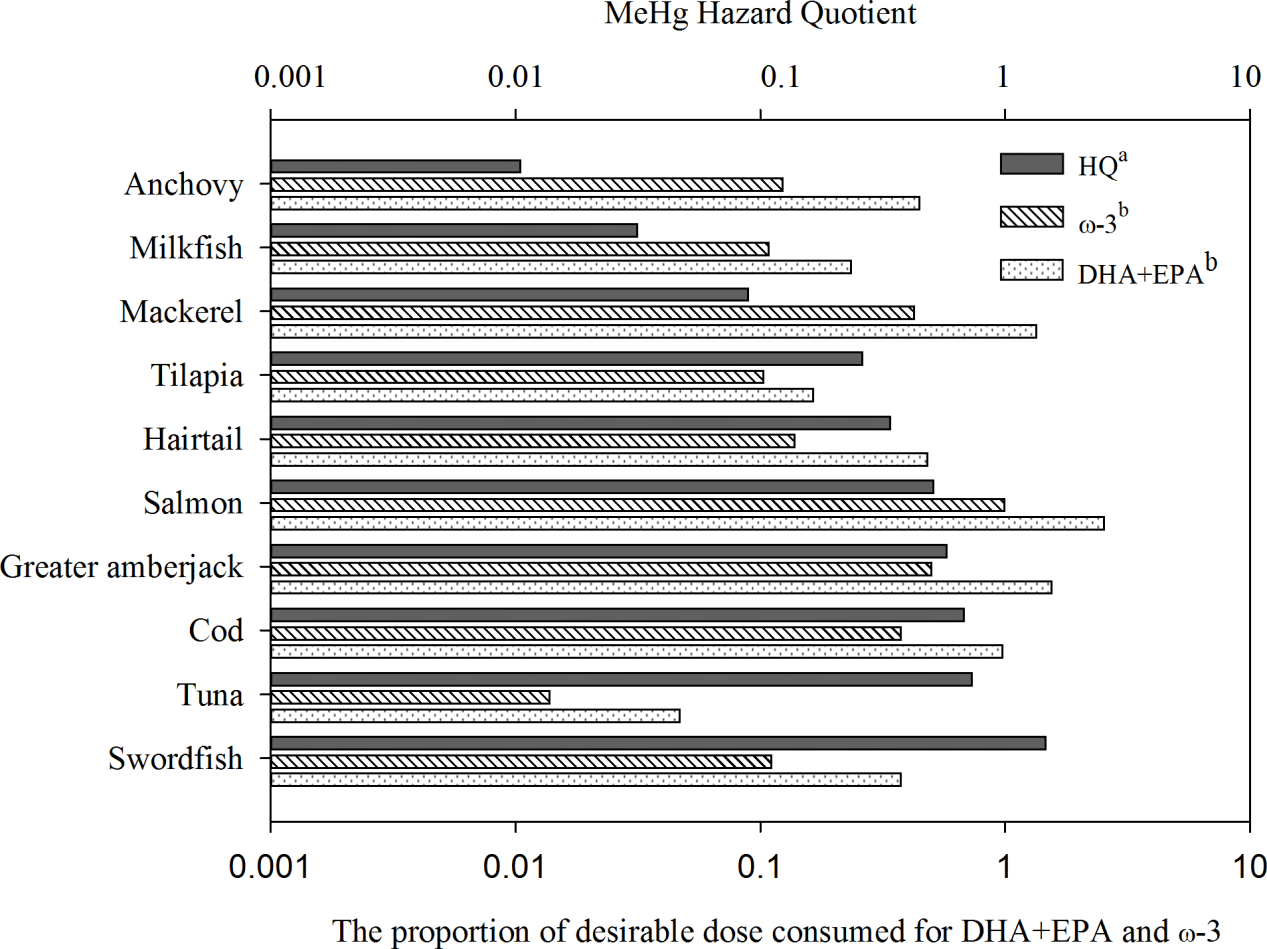
Estimated hazard quotient (HQ) values and the proportion of desirable dose consumed for DHA + EPA and ω-3 PUFA concentrations of specific fish.

An evaluation of the risks of MeHg as well as the benefits of ω-3 PUFA and DHA + EPA from specific fish intake is critical for fish advisories and recommendations. Fish is the most critical source of ω-3 PUFA, and it accumulates EPA and DHA through a trophic chain through marine phytoplankton [43]. Predatory fish could accumulate more MeHg compared with small fish through bioaccumulation mechanisms. Therefore, the risks and benefits to women resulting from MeHg and fatty acid intake through fish consumption were subsequently examined in our study. To perform the risk–benefit assessment, data pertaining to fatty acid concentrations were first obtained from the Taiwan Food and Drug Administration, as listed in Table 3. Fig 3 displays the results of the estimated HQ and proportion of desirable dose consumed for the DHA + EPA and ω-3 PUFA concentrations of specific fish. The HQ values ranged from 0.01 to 1.50. A HQ value below 1 for a specific fish typically indicates that the health risks from MeHg exposure are not potentially concerning, except for swordfish. Three fish species, namely salmon, mackerel, and greater amberjack, can provide sufficient desirable doses for DHA + EPA consumption.

**Table 3:** Fatty acid concentrations (mg/g wet wt.) and MeHg concentration (mg/kg wet wt.) in the 10 most popular fish consumed in markets in Taiwan^a^.

	Anchovy	Milkfish	Mackerel	Tilapia	Hairtail	Salmon	Greater amberjack	Cod	Tuna	Swordfish
SFA^b^	3.3	45.6	15.2	9.3	7.9	31.2	14.3	5.3	0.3	4.3
MUFA^c^	1.1	48.8	13.6	16.0	6.2	64.8	11.4	125	0.2	2.3
PUFA^d^	4.4	23.1	15.1	9.4	5.0	41.2	17.8	14.9	0.5	4.1
ω-3^e^	4.2	3.7	14.5	3.5	4.7	33.9	17.1	12.8	0.47	3.8
EPA^f^	1.0	0.3	3.1	0.2	0.9	8.9	3.2	4.4	0.1	0.2
DHA^g^	2.8	1.7	8.3	1.2	3.2	12.7	10.0	3.9	0.3	3.0
MgHg (mg/kg wet wt.)	0.002	0.006	0.017	0.050	0.065	0.097	0.110	0.130	0.140	0.280

^a^ Data from the Taiwan Food and Drug Administration; https://consumer.fda.gov.tw/Food/TFND.aspx?nodeID=178

^b^ Saturated fatty acid

^c^ Monounsaturated fatty acid

^d^ Polyunsaturated fatty acid

^e^ ω-3 fatty acid

^f^ Eicosapentaenoic acid

^g^ Docosahexaenoic acid

**Fig 4:**
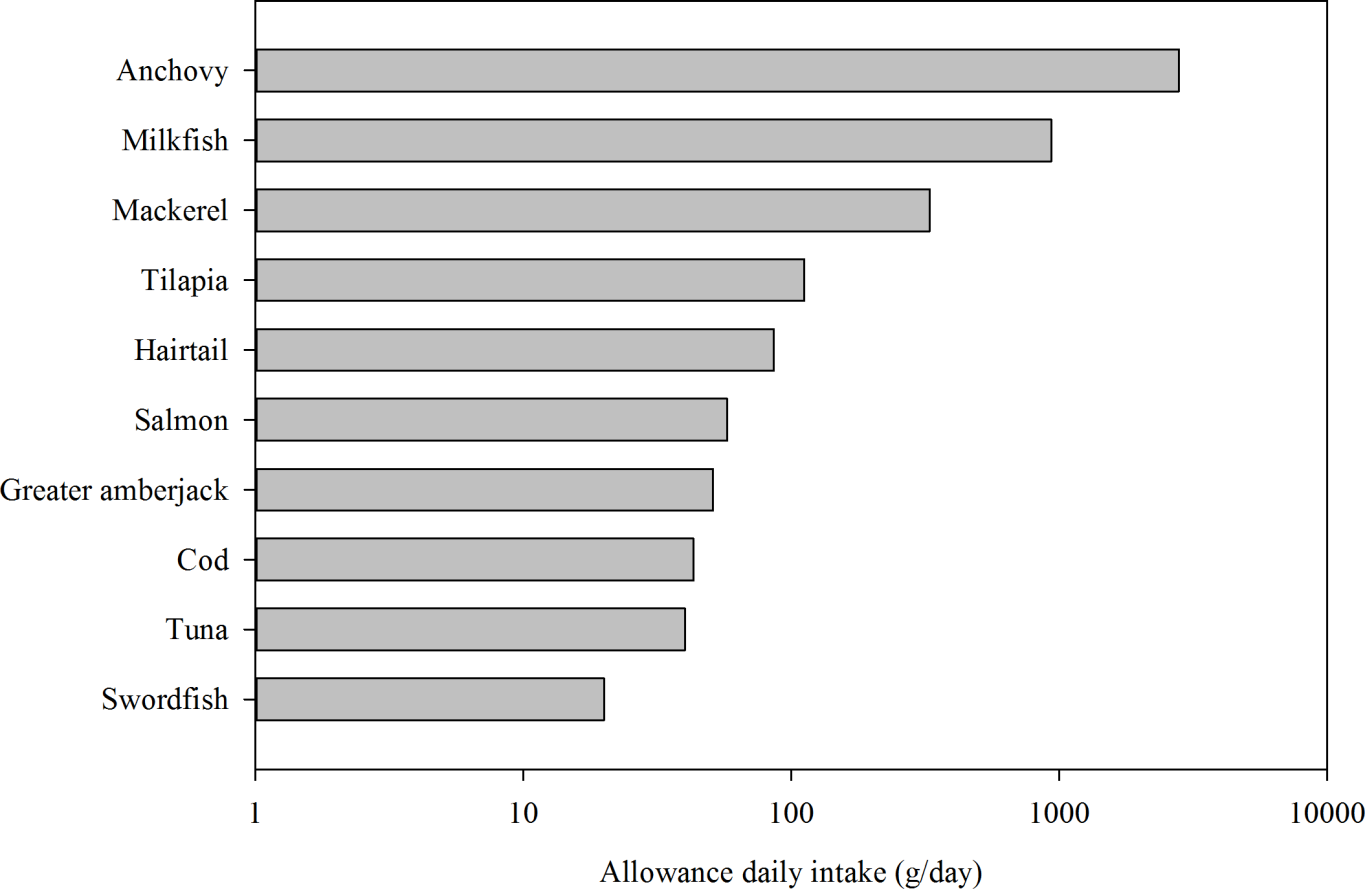
Allowance of daily MeHg intake from specific fish consumption.

Fig 4 shows the allowance daily MeHg intake of specific fish. According to the reference dose of the US Environmental Protection Agency and the MeHg concentration of specific fish, the allowable daily intake ranged from 20 to 2800 g/d. In our study, the allowable daily intake was a measure of the amount of MeHg in fish that can be ingested daily over a lifetime without an appreciable risk of deleterious health effects. The estimated daily fish intake to reach the minimum recommended value of DHA + EPA and ω-3 PUFA ranged from 15.7 to 690 g/d and from 30 to 2127 g/d, respectively (data not shown). Previous studies showed that the EPA concentration in mackerel ranged from 2.2 to 14.5 mg/g (wet wt.), and in salmon, it ranged from 6.4 to 13.0 mg/g (wet wt.); DHA in mackerel ranged from 2.8 to 21.6 mg/g (wet wt.), whereas salmon contained DHA between 5.6 and 17.0 mg/g (wet wt.) [44, 45]. In the current study, several species of salmon, mackerel, and greater amberjack had high ω-3 PUFA, EPA, and DHA content, but their average MeHg level was from 0.017 to 0.110 mg/kg. Conversely, another 2 fish species, swordfish and tuna, with average MeHg levels of 0.14 to 0.28 mg/kg, were not particularly rich sources of ω-3 PUFA, EPA, and DHA. These results were consistent with those of previous studies and further suggested that tuna and swordfish contain substantially greater concentrations of MeHg and lower concentrations of DHA + EPA and ω-3 PUFA compared with other fish species. By contrast, ingestion of mackerel and salmon, which have high concentrations of DHA + EPA and ω-3 PUFA, could promote optimal health for women. These results also revealed that fish at lower trophic levels had a low concentration of MeHg but relatively high DHA + EPA and ω-3 PUFA content. Oken et al. [46] reported that infants from U.S. mothers with maternal fish consumption of > 2 servings per week and a hair Hg level of ≤ 1.2 mg/kg could obtain a higher cognition score. Mother could continue to eat fish during pregnancy but should select fish with lower Hg content, which was associated with an increased cognition score. DHA in fish has been known to be an essential dietary nutrient for rapid brain growth from the third trimester to 2 years of age and is transported mainly via the placenta to the fetus [47–50]. Thus, maternal DHA and ω-3 PUFA intake through fish consumption has also been considered critical factors that may influence prenatal neurodevelopmental outcomes.

Although the potential influence of MeHg intake through specific fish consumption by women was inferred in this study, women of childbearing age may also be exposed to other environmental contaminants such as polybrominated diphenyl ethers and polychlorinated biphenyls through fish intake [51, 52]. Collecting additional data in future research pertaining to the intake of MeHg as well as other critical environmental pollutants and fatty acids is very crucial for issuing further recommendations for women of childbearing age regarding fish consumption.

## Conclusion

In this study, we established a correlation between hair MeHg concentrations and daily MeHg exposure among pregnant and infertile women. Approximately 80% of the infertile women and 68% of the pregnant women had hair MeHg concentrations that exceeded the reference dose of 1 mg/kg established by the US Environmental Protection Agency. Hg may influence the endocrine system, which could lead to a decrease in ovarian function and impair fertility. We also found that the estimated daily MeHg intake from fish was significantly correlated with MeHg concentration in hair. The daily MeHg exposure dose for infertile women was significantly greater than that for pregnant women. Thus, MeHg could be one of the risk factors that may affect fertility. The risks of MeHg and the benefits of ω-3 PUFA and DHA + EPA from fish intake were further evaluated and discussed according to their mean concentrations in fish. The MeHg concentrations in fish in our study did not exceed the Codex guideline level of 1 mg/kg. However, the choice of which fish to consume was shown to be a critical factor for MeHg exposure and ω-3 PUFA and DHA + EPA intake in women. Our results revealed that salmon, mackerel, and greater amberjack could be healthy fish species to consume for women of childbearing age because of their low MeHg concentration and high concentration of DHA + EPA and ω-3 PUFA.

## Supporting Information

S1 Table Hair Hg concentrations in pregnant women (n = 32). (DOC)

S2 Table The MeHg concentrations (mg/kg wet wt.) in the 10 most popular fish muscles for woman of childbearing age. (DOC)
