# Supplementary material for: Methylmercury Concentration in Fish and Risk-Benefit Assessment of Fish Intake among Pregnant versus Infertile Women in Taiwan
Source: PLoS One. 2016 May 17;11(5):e0155704. doi: 10.1371/journal.pone.0155704 (PMC4871344; doi:10.1371/journal.pone.0155704)
Supplement: S1 Table — (DOC) [file pone.0155704.s001.doc]

**S1 Table. Hair Hg concentrations in pregnant women (n=32).**

|  | Hair Hg concentration (mg/kg) | | | |
| --- | --- | --- | --- | --- |
|  | Mean ± SE^a^ | Geomean (GSD) | Median | p – value^b^ |
| First trimester | 1.54 ± 0.78 | 1.35 (0.15) | 1.31 | 0.54 |
| Second trimester | 1.74 ± 1.50 | 1.39 (0.27) | 1.33 |  |
| Third trimester | 1.56 ± 0.95 | 1.34 (0.17) | 1.45 |  |

^a^ standard error

^b^ statistical test: ANOVA
