# Supplementary material for: Methylmercury Concentration in Fish and Risk-Benefit Assessment of Fish Intake among Pregnant versus Infertile Women in Taiwan
Source: PLoS One. 2016 May 17;11(5):e0155704. doi: 10.1371/journal.pone.0155704 (PMC4871344; doi:10.1371/journal.pone.0155704)
Supplement: S2 Table — (DOC) [file pone.0155704.s002.doc]

**S2 Table. The MeHg concentrations (mg/kg wet wt.) in the 10 most popular fish muscles for woman of childbearing age.**

| Species | Scientific name | n | Length (cm) | Weight (g) | MeHg (mg/kg) | Edible proportion | Feeding habits |
| --- | --- | --- | --- | --- | --- | --- | --- |
|  |  |  |  |  | (Mean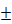SD) | (%) |  |
| Anchovy larvae | *Stolephorus commersonnii* | 3 | 1.83±0.28 | 0.50±0.21 | 0.002±0.001 | 100 | Filter-feeding |
| Milkfish | *Chanos chanos* | 3 | 19.1±0.64 | 163±24.3 | 0.006±0.01 | 67.0 | Omnivorous |
| Mackerel | *Scomber australasicus* | 3 | 27.2±4.01 | 261±92.5 | 0.017±0.03 | 15.2 | Carnivorous |
| Tilapia | *Oreochromis mossambicus* | 3 | 24.7±6.53 | 534±231 | 0.050±0.06 | 38.9 | Carnivorous |
| Hairtail (fillet) | *Trichiurus lepturus* | 3 | - | - | 0.065±0.06 | 60.0 | Carnivorous |
| Salmon (fillet) | *Oncorhynchus mykiss* | 3 | - | - | 0.097±0.14 | 79.3 | Carnivorous |
| Greater amberjack (filet) | *Seriola dumerili* | 3 | - | - | 0.110±0.08 | 57.0 | Carnivorous |
| Cod (fillet) | *Gadus macrocephalus* | 3 | - | - | 0.130±0.05 | 81.4 | Carnivorous |
| Tuna (fillet) | *Thunnus alalunga* | 3 | - | - | 0.140±0.13 | 100 | Carnivorous |
| Swordfish(fillet) | *Xiphias gladius* | 3 | - | - | 0.280±0.23 | 100 | Carnivorous |
